# Impact of video-led educational intervention on uptake of influenza vaccine among the elderly in western China: a community-based randomized controlled trial

**DOI:** 10.1186/s12889-022-13536-8

**Published:** 2022-06-06

**Authors:** Minghuan Jiang, Xuelin Yao, Pengchao Li, Yu Fang, Liuxin Feng, Khezar Hayat, Xinke Shi, Yilin Gong, Jin Peng, Naveel Atif

**Affiliations:** 1 Department of Pharmacy Administration and Clinical Pharmacy, School of Pharmacy, Xi’an Jiaotong University, Xi’an, 710061 China; 2 Center for Drug Safety and Policy Research, Xi’an Jiaotong University, Xi’an, 710061 China; 3 Shaanxi Center for Health Reform and Development Research, Xi’an, 710061 China; 4 Research Institute for Drug Safety and Monitoring, Institute of Pharmaceutical Science and Technology, Western China Science & Technology Innovation Harbor, Xi’an, 712000 China; 5 Department of Pharmacy, The Second Affiliated Hospital of Xi’an Jiaotong University, Xi’an, 710004 China; 6 Health Science Center, Xi’an Jiaotong University, Xi’an, 710061 China

**Keywords:** Influenza vaccine, Vaccination coverage, Randomized controlled trial, Awareness, Elderly

## Abstract

**Background:**

Influenza vaccination coverage rate among the elderly is low in China. We aimed to evaluate the impact of video-led educational intervention on influenza vaccine uptake among the Chinese elderly.

**Methods:**

A randomized controlled trial was conducted in 8 communities of Xi’an, a representative city in western China. Elderly aged over 60 years were randomized to the control group and intervention group (12-minute video education on influenza and its vaccination). Participants’ knowledge, attitudes, and practices (KAP) of influenza was assessed by using a questionnaire survey before and after the intervention. The primary outcomes were participants’ willingness to get influenza vaccinated and their actual uptake rates in the 2020-21 flu season. Secondary outcomes were the variations of pre- and post-intervention KAP scores. Intention-to-treat analysis was performed to analyze the data, and sensitivity analyses were conducted to examine the robustness of the results.

**Results:**

A total of 350 people were enrolled, with 175 individuals for each group. Participants in the intervention group were more willing to receive influenza vaccination than those in the control group (64.6% vs. 51.4%, *p*<0.05). The influenza vaccination uptake rate occurred in 10.3% of participants in the intervention group and 3.4% in the control group (odds ratio, 3.23; 95% CI 1.25-8.32, *p<*0.001). The post-intervention KAP scores in the intervention group were significantly higher compared to those in the control group (*p<*0.001).

**Conclusion:**

Video-led education was an effective and feasible approach to improve old people’s willingness and uptake of influenza vaccination in western China.

**Supplementary Information:**

The online version contains supplementary material available at 10.1186/s12889-022-13536-8.

## Background

Influenza tends to cause an epidemic among individuals globally, and the influenza pandemic was proposed as one of the ten threats to human health by the World Health Organization (WHO) [1]. Influenza leads to 290,000-640,000 deaths around the world annually, with 84%-95% of deaths occurring in old people [2–4]. With the rapidly aging population in China [5], influenza-related cases and complications could be a substantial disease burden for the old people. The influenza vaccine is regarded as the most effective approach to prevent influenza infection. The Technical Guidelines for Seasonal Influenza Vaccination in China (2020-2021) [6] and Health China Campaign 2019-2030 [7] both recommended that the elderly aged over 60 years were one of the priority groups to get influenza vaccination.

Influenza vaccine is not included in the National Immunization Program in China, while some developed regions provided fully or partial reimbursement for high-risk population receiving vaccination. Influenza vaccines are widely available in community or township health centers, however, the coverage of influenza vaccination among Chinese old people was extremely low (4%) [8] compared to the 75% vaccination target recommended by WHO [9]. Lack of vaccine-related knowledge, suspicion of vaccine safety, and low vigilance of influenza-related risks were the main barriers of influenza vaccination in the elderly [10, 11]. A recent meta-analysis with studies in Asian populations such as Hong Kong reported that interventions by physicians’ reminders, home visits, face-to-face conversation, clinic posters, and free influenza vaccine could result in a 1.11 to 152.95-fold increase in influenza vaccine uptake among old people over 60 years [12].

Previous studies reported that knowledge and understanding were the main barriers of expanding influenza vaccination in Chinese old people [13–15]. Educational interventions were found to be one of the most effective approaches to improve vaccine coverage via oral communication, posters, brochures, videos, or surface mails [16–19]. Compared to brochures, emails, or text messages, videos have proved more acceptable and intuitive due to its compound elements with images, words, and sounds making it easier to understand, especially for the elderly [20]. To our knowledge, there have been no prior studies on video-led educational interventions in elderly Chinese to explore its usefulness in influenza vaccination. Since the influenza vaccination rate was lowest in western regions of China [21], the present study aimed to assess the impact of video-led educational intervention on influenza vaccine uptake among old people in western China.

## Methods

### Participants and randomization

We designed a community-based randomized controlled trial (RCT) to perform the present study, which was prospectively registered at the Chinese Clinical Trial Registry (ChiCTR2000034330) on 02/07/2020. A detailed research protocol was reported elsewhere previously [22]. In short, we selected Xi’an, the capital city of Shaanxi province, as the representative region of western China. Based on the average gross domestic product per capita in all Xi’an districts, we randomly selected Yan Ta and Xin Cheng as developed districts and Chang An and Ba Qiao as underdeveloped districts [23]. Two community healthcare centers in each district were selected by using computer-generated random numbers. Participants were therefore randomly recruited from old adults with regular medical check-ups based on health records in 8 community healthcare centers. Participants were informed about the significance of the study, and those who agreed to participate in were randomized to the intervention and control groups, respectively, in a 1:1 ratio. The inclusion criteria of eligible participants were: 1) elderly aged 60 years or above; 2) living in the community for at least one year; 3) willing to participate in this study with an informed consent form. Individuals with hearing or eye-sight problems, cognitive disorders, or serious psychological illnesses were excluded from the study.

The uptake rate of influenza vaccine among old people in western China was estimated to be 2% [21]. We assumed that the post-intervention uptake rate would have a 5-fold increase to 10% in our study. With the power of 0.9 and significance level of 0.05, it required 80 participants in each group according to a Chi-square test in the G-power statistical analysis program [24]. If the lost follow-up rate of participants was 20%, the sample size in each group was calculated to be a minimum of 96 in the present study.

### Video-led educational intervention

Two post-graduate medical students designed the content of the education video; thereafter we invited experts from the Center for Disease Control and Prevention and Public Hospitals in Xi’an to provide recommendations to upgrade the video. A pilot test was conducted on ten old people to improve the acceptability and visualization of the video. The final 12-minute video was composed of 1) knowledge of influenza, including its epidemic risks, influenza-related complications, transmission, and prevention of influenza infection; 2) information on the influenza vaccine, including its safety and effectiveness, time for vaccination, and vaccination recommendations; 3) several cases of influenza pandemic globally to illustrate the severity of influenza diseases.

### Data collection

To assess the effect of the intervention, a questionnaire survey on participants’ knowledge, attitudes, and practice (KAP) about influenza and the vaccine was performed before and after the intervention. The questionnaire (Additional file 1) consisted of four sections: 1) socio-demographic characteristics of the participants, including gender, age, monthly income, occupation, education, condition of chronic diseases, and history of influenza vaccination in the previous season; 2) knowledge of influenza and influenza vaccine; 3) attitudes toward influenza vaccination; 4) practices to receive influenza vaccination. We conducted a pilot survey with 30 old people to evaluate the questionnaire validity, and the Cronbach alpha coefficient of the questionnaire was 0.844. The primary outcomes assessed in the present study were participants’ willingness to get vaccinated and the uptake rate of influenza vaccine. Secondary outcomes were the variations of participants’ KAP scores.

We recruited 10 medical students from Xi’an Jiaotong University to conduct the present study. They were well-trained on the objectives of our study, the process of intervention, and communication skills with old people. The study was performed from September to November 2020 before the influenza outbreak in the 2020-21 season. From the beginning of September to the end of October, participants in the intervention group received a basic medical examination and a 12-minute video education on influenza and influenza vaccines at the healthcare centers. Subsequently, participants had a 15-minute group discussion on the content of the video, and medical students were responsible for answering their questions raised from the video. Participants in the control group only received a basic medical examination. At the end of November, the medical students called all participants to ask and record their vaccination status and test their KAP scores using the same questionnaire.

### Statistical analysis

Intention-to-treat analysis was performed to analyze data using Microsoft Excel 2010 and IBM SPSS 21.0 [25]. Participants who were lost follow-up were considered unvaccinated and reluctant to be vaccinated. The post-intervention data on them were consistent with their data in baseline before intervention. To examine the robustness of our results, we also conducted sensitivity analysis by excluding data from participants who were lost follow-up. Findings on participants’ socio-demographic characteristics, uptake of influenza vaccine, and their willingness to get vaccinated were presented by descriptive statistics by proportion. Their KAP scores were shown using a mean with a standard deviation. Chi-square test and Wilcoxon rank-sum test were used to evaluate the difference of participants’ characteristics and KAP scores in baseline, respectively. A Chi-square test was performed to compare the difference in post-intervention willingness to get vaccination and vaccine uptake in two groups. T-test was used to compare the variation of pre-and post-intervention KAP scores. Multivariate logistic regression analysis was performed to determine the association between educational intervention and uptake of influenza vaccine. Participants’ socio-demographic characteristics including gender, age, occupation, educational level, monthly income, status of chronic diseases, and vaccination in the previous season were adjusted. *P*<0.05 was considered statistically significant. All methods were carried out in accordance with relevant guidelines and regulations.

## Results

In total, 400 people were recruited from 8 communities. 24 (6%) individuals refused to participant in the study, 15 (3.75%) individuals did not meet the inclusion criteria, and 11 (2.75%) did not complete the pre-intervention questionnaire survey. Therefore, 350 participants with 175 old people were enrolled in the RCT for each group. Participants’ baseline socio-demographic characteristics in the two groups showed no significant difference (Table 1). The mean vaccination rates in the previous flu season were 3.4% in the intervention group and 2.9% in the control group (*p*=0.759). The proportion of participants who were willing to get vaccinated in the future was 44.6% and 46.9% in the intervention and control groups, respectively (*p*=0.912). The pre-intervention KAP scores also showed no significant difference in the two groups.

**Table 1:** Participants’ socio-demographic characteristics in baseline

	**Control group** ***N* (%)**	**Intervention group** ***N* (%)**	***P-*value**
Gender			0.830
Male	78 (44.6)	80 (45.7)	
Female	97 (55.4)	95 (54.3)	
Age (years)			0.440
60-69	85 (48.6)	90 (51.4)	
70-79	77 (44.0)	67 (38.3)	
≥80	13 (7.4)	18 (10.3)	
Occupation			0.859
Retirement	81 (46.3)	79 (45.1)	
Full-time/part-time job	15 (8.6)	18 (10.3)	
Other	79 (45.1)	78 (44.6)	
Education level			0.676
Primary school or below	61 (34.9)	65 (37.1)	
High school	86 (49.1)	78 (44.6)	
College or above	28 (16.0)	32 (18.3)	
Monthly income (Chinese Yuan)			0.426
≤1,000	93 (53.1)	83 (47.4)	
1,000-4,000	57 (32.6)	59 (33.7)	
≥4,000	25 (14.3)	33 (18.9)	
Chronic diseases			0.442
Yes	111 (63.4)	104 (59.4)	
No	64 (36.6)	71 (40.6)	
Get vaccinated in previous season			0.795
Yes	5 (2.9)	6 (3.4)	
No	170 (97.1)	169 (96.6)	
Willing to get vaccinated in future			0.912
Yes	82 (46.9)	78 (44.6)	
No	48 (27.4)	50 (28.6)	
Uncertain	45 (25.7)	47 (26.8)	
Awareness of influenza			
Knowledge score (0-10)	5.71±2.02	5.69±1.61	0.588
Attitude score (8-40)	27.19±4.04	27.44±3.13	0.611
Practice score (0-12)	9.13±1.44	9.24±1.27	0.679

**Fig. 1:**
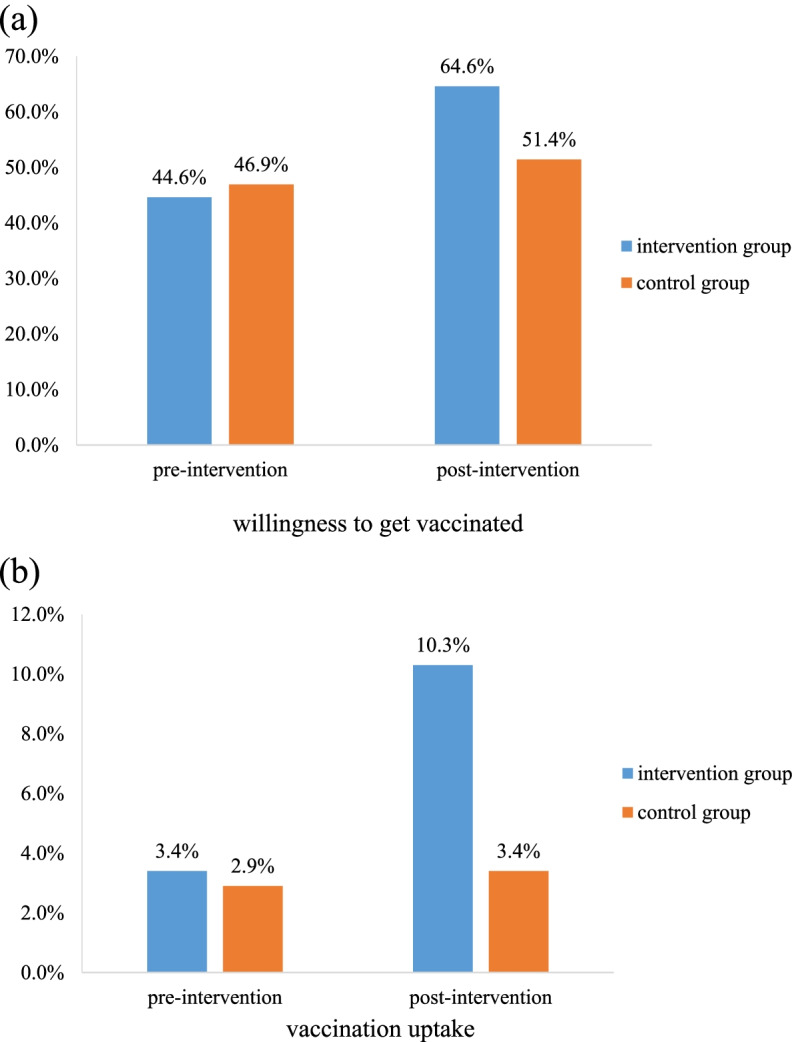
Pre- and post-intervention (**a**) willingness to get vaccinated, and (**b**) uptake of influenza vaccine.

The results of intention-to-treat analysis for the present study were shown in Fig. 1. Participants in the intervention group were more willing to receive influenza vaccination than those in the control group (64.6% vs. 51.4%, *p*<0.05). The influenza vaccination uptake rate occurred in 10.3% of participants in the intervention group and 3.4% in the control group (unadjusted odds ratio, 3.23; 95% confidence interval (CI): 1.25-8.32, *p*<0.001). Although the uptake rate in the intervention group was numerically higher (not statistical difference) than that in the control group before intervention, the variation of vaccination rates between two groups was found to be statistically different. The variations of participants’ pre-and post-intervention KAP scores in both groups were shown in Table 2. All KAP scores in the intervention group increased significantly compared to those in the control group post-intervention (*p<*0.001). The logistic regression analysis found that monthly income, educational intervention, and knowledge and practice scores were significant predictors of vaccine uptake (Table 3). The vaccine uptake rate of participants receiving the educational intervention was 3.63-fold higher compared to those who were in the control group (adjusted odds ratio, 3.63; 95%CI: 1.31-10.06; *p*=0.013).

**Table 2:** Variation of participants’ pre- and post-intervention KAP scores

	**Pre-intervention (Mean ± SD)**			**Post-intervention (Mean ± SD)**		
	**Control group**	**Intervention group**	***P*-value**	**Control group**	**Intervention group**	***P*-value**
Knowledge score	5.71±2.02	5.69±1.61	0.588	5.88±1.76	6.51±1.58	<0.001
Attitude score	27.19±4.04	27.44±3.13	0.611	27.25±4.03	29.21±3.58	<0.001
Practice score	9.13±1.44	9.24±1.27	0.679	9.13 ±1.48	9.39±1.26	<0.001

*KAP* knowledge, attitudes, and practice, *SD* standard deviation.

**Table 3:** Significant predictors of vaccine uptake in logistic regression analysis

	Uptake rate (%)	Odds ratio (95%CI)	*P-*value
Monthly income (Chinese Yuan)			
≤1,000	3.1	-	
1,000-4,000	9.6	5.45 (1.78-16.64)	0.003
≥4,000	10.8	5.17 (1.47-18.22)	0.011
Educational intervention			
Control group	3.4	-	
Intervention group	10.3	3.63 (1.31-10.06)	0.013
Knowledge	-	1.41 (1.03-1.94)	0.034
Practice	-	2.15 (1.33-3.48)	0.002

Logistic regression models were adjusted for participants’ gender, age, occupation, educational level, monthly income, status of chronic diseases, and vaccination in previous season.

In our study, 32 and 31 participants were lost to follow-up in the intervention and control groups, respectively. To examine the robustness of above findings, sensitivity analysis was performed by excluding data from these participants. The findings for primary and secondary outcomes were consistent with results as shown in the intention-to-treat analysis (Table 4). In summary, participants’ willingness to get vaccinated, the uptake of influenza vaccine, and the post-intervention KAP scores in the intervention group were significantly higher than those in the control group.

**Table 4:** Results of sensitivity analysis after intervention

	**Control group** **(*N*=144)**	**Intervention group** **(*N*=143)**	***P*-value**
Willingness to get vaccinated (%)	80 (55.6)	99 (69.2)	<0.001
Uptake of influenza vaccine (%)	6 (4.2)	18 (12.6)	<0.001
Knowledge score (mean ± SD)	6.03±1.56	6.92±1.35	<0.001
Attitude score (mean ± SD)	27.86±3.80	29.74±2.67	<0.001
Practice score (mean ± SD)	9.64±1.02	9.72±0.81	0.016

*SD* standard deviation.

## Discussion

We performed a RCT study and found that video-led educational intervention had a positive effect on the uptake of influenza vaccine among old people in China. The impact included participants’ willingness to get vaccinated and the actual uptake of the influenza vaccine significantly increased by 20.0 and 6.9 percentage points by the intervention, respectively. There were no statistically significant changes in the control group. The uptake rate was significantly associated with the improved knowledge and practice scores of old people.

The significant increase in influenza vaccination after intervention in our study was consistent with the impact of previous educational approaches [16, 26]. A study in eastern China examined the effectiveness of implementing the recommendation for influenza vaccination from community healthcare workers to elderly people with chronic diseases [27]. The results showed that the vaccination coverage increased from 0.3% to 19% after the intervention. Another study from Hong Kong reported that the influenza vaccination rate among the elderly increased 34% by using 3-min face-to-face verbal health education and a pamphlet regarding influenza [26]. However, the absolute post-intervention uptake rate (10.3%) in our study was still far below the target of 60-80% to achieve herd immunity [28]. Therefore, multiple interventions could be combined to increase further the uptake of influenza vaccine in China, such as expanding vaccine access, financial incentives for vaccination, national or regional immunization programs, or personal advocacy of vaccination by community-based home visits [12].

In the present study, although nearly half of the participants (45.7%) were willing to get the influenza vaccination, the pre-intervention uptake (3.14%) was far below expected. A study in Shanghai showed similar findings that the willingness to self-pay for influenza vaccine was 70.5% among Chinese aged 50 to 59 years, while the actual uptake was 10.8% [29]. The great gap between willingness and vaccination behavior was possibly associated with vaccine hesitancy [11]. Factors of cost, convenience or access to vaccines were possible barriers of vaccine hesitancy that prevent those who willing to be vaccinated from actual vaccination. A meta-analysis of intention-behavior tests showed that median-to-big change of intention yielded a small-to-median change of behavior [30]. Therefore, the increase of post-intervention vaccination uptake in our study could be partly explained by participants’ vaccination willingness increasing from 44.6% to 64.6%.

The KAP scores improved significantly among old people who received video-led educational intervention in our study. The information delivered by the video served as an impetus for more positive beliefs towards influenza vaccine for the elderly. Individuals’ awareness towards influenza vaccination were facilitators of vaccine uptake [10, 11]. Unvaccinated people had low knowledge score on the severity of influenza and the effectiveness and safety of the vaccine [10]. A similar study from Singapore reported that old peoples’ knowledge gaps on influenza would affect their attitudes towards vaccination uptake [31]. A community-based educational intervention could improve knowledge scores across genders, ages, and educational levels. Previous studies on the evaluation of adult KAP related to influenza and vaccine in China reported that most people viewed influenza as a common disease, and its threat to society was low [32, 33]. They agreed influenza could not lead to severe health and economic burden; therefore, they did not get vaccinated in practice.

Besides personal beliefs towards getting a vaccination, social factors also influenced the regional or national vaccine uptake rate, including recommendations from healthcare professionals or immunization programs for vaccination [31]. Introducing a vaccination program from a healthcare system or societal perspective may greatly influence old people’s behavior on vaccination uptake. To date, the influenza vaccine has not been included in the national immunization program in China. Around 61 regions in China provided fully or partial reimbursement for high-risk populations (old people, children, healthcare workers) receiving influenza vaccination [34]. The reimbursement of influenza vaccination was a motivator for uptake of influenza vaccination. For instance, the vaccination rate increased from 1.7% to 38.7% among old people after implementing a free vaccination program in Beijing [13]. However, the increased vaccination rate was still lower than in some developed countries with similar vaccination policies [35].

There were several limitations in the present study. Firstly, we selected Xi’an as the representative city in western China and samples may be biased towards those who were willing to participate and get immunized in our study, which might also limit the generalization of our findings to the whole nation. In future, the interventional strategies recommended by the WHO Europe office Tailoring Immunization Programs could be adopted to examine their effects on vaccine uptake from a country perspective [36], which have proved great success after implementation in some developed countries [37]. Secondly, we performed the intervention before the 2020-21 flu season and assessed its outcome at the end of the following month, which might underestimate the long-term impact of the video-led educational intervention. The post-intervention uptake of the influenza vaccine in this season among the elderly could be possibly higher than the data reported in our study. Thirdly, the end outcomes of vaccination uptake and willingness were reported by the participants. Their actual vaccination were not available since we were not able to access to their medical records. Social desirability bias might exist in both groups after intervention, especially in the intervention group. Lastly, the actual vaccination rates in both groups might be influenced by the unexpected shortage of influenza vaccines in the 2020-21 flu season because the coronavirus disease epidemic of 2019 (COVID-19) increased the demand for influenza vaccines in China [38]. The COVID-19 might also influence participants’ perceptions of knowledge and attitude towards influenza in both groups [33]. Although we were not able to assess its exact impact on our findings, we conducted rigorous sensitivity analysis to examine the robustness of our results and the effect of our intervention.

## Conclusion

Despite a short-term duration of intervention in our study, video-led education achieved a positive impact and proved to be an effective approach to increase old people’s willingness and uptake of influenza vaccination in western China. We recommended that policymakers further justify the cost-effectiveness of generalized application to broader populations and regions in China.

## Supplementary Information

**Additional file 1.** Questionnaire for survey.

## Data Availability

The datasets used and/or analysed during the current study are available from the corresponding author, subject to approval from the ethics committee that approved the original study.

## Abbreviations

WHO: World Health Organization
RCT: Randomized controlled trial
KAP: Knowledge, attitudes, and practice
