# Supplementary material for: Impact of video-led educational intervention on uptake of influenza vaccine among the elderly in western China: a community-based randomized controlled trial
Source: BMC Public Health. 2022 Jun 6;22:1128. doi: 10.1186/s12889-022-13536-8 (PMC9169441; doi:10.1186/s12889-022-13536-8)
Supplement: Supplementary file 1 — Additional file 1. Questionnaire for survey. [file 12889_2022_13536_MOESM1_ESM.docx]

**Additional file 1 Questionnaire for survey**

**Basic information**

1. Gender: □Male □Female
2. Age (years): □60-69 □70-79 □≧80

3. Occupation: □Retirement □Full-time job/Part-time job □others______

4. Education: □Primary school and below □Secondary school □College school and above

1. Monthly income (RMB): □<1000 □1000-4000 □>4000

6. Chronic disease: □None □One and above

**Knowledge**

1. There is a difference between the flu and cold.

□Yes □No □Unclear

1. Influenza is a respiratory disease caused by the influenza virus.

□Yes □No □Unclear

1. Influenza is infectious.

□Yes □No □Unclear

1. Influenza is spread mainly by droplets of respiratory secretions.

□Yes □No □Unclear

1. Influenza will bring some complications, such as pneumonia, otitis media.

□Yes □No □Unclear

1. Influenza is mainly divided into type A and type B.

□Yes □No □Unclear

1. Influenza vaccine can prevent influenza effectively.

□Yes □No □Unclear

1. Influenza vaccine needs to be vaccinated annually.

□Yes □No □Unclear

1. The elderly above sixty years old is one of the groups who should receive the influenza vaccine preferentially.

□Yes □No □Unclear

1. Influenza vaccine is divided into trivalent and the quadrivalent influenza vaccine.

□Yes □No □Unclear

**Attitude**

1.The elderly are at higher risk than a common adult of being attacked by flu.

□Strongly agree □Agree □Uncertain □Disagree □Strongly disagree

2.There is a severe disease and economic burden when the elderly have influenza.

□Strongly agree □Agree □Uncertain □Disagree □Strongly disagree

3.Influenza can be treated by antibiotics like amoxicillin and cephalosporins.

□Strongly agree □Agree □Uncertain □Disagree □Strongly disagree

4.There is no need to receive an influenza vaccine for the elderly.

□Strongly agree □Agree □Uncertain □Disagree □Strongly disagree

5.Influenza vaccine is safe.

□Strongly agree □Agree □Uncertain □Disagree □Strongly disagree

6.Influenza won’t occur after receiving the influenza vaccine.

□Strongly agree □Agree □Uncertain □Disagree □Strongly disagree

7.Influenza vaccination should be free.

□Strongly agree □Agree □Uncertain □Disagree □Strongly disagree

8.The community should strengthen education and knowledge propaganda about the prevention of influenza.

□Strongly agree □Agree □Uncertain □Disagree □Strongly disagree

**Practice**

1. Do you take measures to prevent influenza during flu season (such as indoor ventilation, wearing a mask when you go out, washing your hands frequently)?

□Yes □No

1. Do you strength physical exercises during flu season?

□Yes □No

1. Will you take part in it if the community holds an educational lecture about influenza?

□Yes □No_______

1. If you have an illness like influenza, you will go for self-medication:

□Yes □No

1. Have you received the influenza vaccine last year (before intervention) **/**recently (after intervention)?

□Yes □No

1. Will you consider influenza vaccination in the future?

□Yes □No
